# The International Classification of Functioning, Disabilities, and Health categories rated as necessary for care planning for older patients with heart failure: a survey of care managers in Japan

**DOI:** 10.1186/s12877-021-02647-3

**Published:** 2021-12-15

**Authors:** Shigehito Shiota, Toshiro Kitagawa, Takayuki Hidaka, Naoya Goto, Naoki Mio, Kana Kanai, Makiko Naka, Hiroko Togino, Mariko Mochizuki, Hiroyuki Ochikubo, Yukiko Nakano, Yasuki Kihara, Hiroaki Kimura

**Affiliations:** 1 Heart Failure Center, Hiroshima University Hospital, Hiroshima, Japan; 2 Division of Clinical Support, Department of Rehabilitation, Hiroshima University Hospital, Hiroshima, Japan; 3 Department of Cardiovascular Medicine, Hiroshima University Graduate School of Biomedical and Health Sciences, Hiroshima, Japan; 4 Department of Nursing, Hiroshima University Hospital, Hiroshima, Japan; 5 Hiroshima Care Manager Association, Hiroshima, Japan; 6 Hiroshima University Graduate School of Biomedical and Health Sciences, Hiroshima, Japan; 7 Department of Rehabilitation Medicine, Hiroshima University Hospital, Hiroshima, Japan

**Keywords:** ICF, Care manager, Heart failure

## Abstract

**Background:**

Establishing an information-sharing system between medical professionals and welfare/care professionals may help prevent heart failure (HF) exacerbations in community-dwelling older adults. Therefore, we aimed to identify the ICF categories necessary for care managers to develop care plans for older patients with HF.

**Methods:**

A questionnaire was administered to 695 care managers in Hiroshima, Japan, on ICF items necessary for care planning. We compared the care managers according to their specialties (medical qualifications and welfare or care qualifications). Furthermore, we created a co-occurrence network using text mining, regarding the elements necessary for collaboration between medical and care professionals.

**Results:**

There were 520 valid responses (74.8%). Forty-nine ICF items, including 18 for body functions, one for body structure, 21 for activities and participation, and nine for environmental factors, were classified as “necessary” for making care plans for older people with HF. Medical professionals more frequently answered “necessary” than care professionals regarding the 11 items for body functions and structure and three items for activities and participation (*p* < 0.05). Medical–welfare/care collaboration requires (1) information sharing with related organisations; (2) emergency response; (3) a system of cooperation between medical care and non-medical care; (4) consultation and support for individuals and families with life concerns, (5) management of nutrition, exercise, blood pressure and other factors, (6) guidelines for consultation and hospitalisation when physical conditions worsen.

**Conclusions:**

Our findings showed that 49 ICF categories were required by care managers for care planning, and there was a significant difference in perception between medical and welfare or care qualifications qualifications.

## Background

In Japan, cardiovascular disease is the second leading cause of death after cancer, accounting for 20.6% of all causes of need for nursing care and 6078.2 billion yen in annual medical costs [1–3]. The Japanese government has developed the Japanese national plan for promotion of measures against cerebrovascular and cardiovascular disease in 2020 [4, 5]. The Japanese national plan includes the establishment of a comprehensive community care system that integrates health, medical and welfare services. Heart failure (HF) is one of the most common cardiovascular diseases, with repeated recurrences and hospital admissions reducing the quality of life of patients and their families, and increasing healthcare costs [6–8]. In Japan, the number of patients with HF continues to increase as the population ages, and it is estimated that the number of cases will exceed 1.3 million by 2030 [9, 10]. In addition, there is an increasing number of older people with HF facing various problems such as frailty, dementia, and living alone. A recent HF registry study in Japan showed that 39.2% of patients with HF were aged ≥85 years, and 21.7% lived alone [11, 12]. Social support and information sharing may prevent rehospitalisation for HF [13, 14], and establishing an information-sharing system between medical and welfare/care professionals in the community is an urgent issue.

The Japanese Heart Failure Society recommends using the International Classification of Functioning, Disabilities, and Health (ICF) for a comprehensive assessment of health conditions and functioning [15]. The ICF aims to provide a uniform, standardized language and conceptual framework for describing health and health-related conditions published by the World Health Organization in 2001. The ICF consists of five components: health status, body function, body structure, activity and participation, environmental factors, and personal factors, and is classified into 362 s-level categories. In Japan, the ICF is expected to be used as a common language between medical and welfare or care professionals. We selected the ICFs necessary for the comprehensive assessment of older people with heart failure in a Delphi survey of medical professionals [16].

In Japan, under the long-term care insurance system, care managers are professionals who prepare care plans for the older people in need of care [17–19]. The qualification as a care manager can be obtained by medical professionals or care workers upon taking the respective exam. Based on the written opinion from the attending physician, the care manager assesses the elderly person in need of nursing care and develops a plan to introduce appropriate nursing care, rehabilitation, and other services. Care plans are developed based on the needs and intentions of the elderly person and their families. Nurses, physical therapists, occupational therapists, and certified care workers provide care and rehabilitation to the elderly according to the care plan created by the care manager. Therefore, for older patients with HF to live in their community, an information-sharing system on HF is important for care managers, based on the ICF.

This study aimed to identify the ICF categories necessary for care managers to develop a care plan and to clarify the elements necessary for medical-care collaboration for older patients with HF.

## Methods

### Participants

We recruited 695 care managers from the Hiroshima Care Manager Association in Japan. Among the study participants, we excluded those who did not consent to the research and did not respond to the questionnaire survey. Hiroshima Prefecture has one of the most standardised population distributions in Japan and is an indicator region for policies regarding older people. Therefore, by targeting care managers in the Hiroshima Prefecture, a reference for the creation of standardised care plans in Japan is anticipated.

### Data collection and measures

The study design employed a questionnaire survey. The survey period was from August to December 2020. We explained the purpose and content of the study to participants at a training session for care managers. The questionnaire was then distributed and the completed questionnaires collected. The questionnaire was the same as the one used in the previous study, a survey study of Registered Instructors of Cardiac Rehabilitation [16]. This questionnaire is based on the ICF checklist with additional ICF categories specific to the older with heart failure according to the opinion of expert panel. This methodology is based on the method used to develop the ICF Core Set [20–23]; however, it was not strictly followed because this study did not intend to create the ICF Core Set. An expert panel consisting of multidisciplinary team members (two cardiologists, two nurses, two physical therapists, one occupational therapist and one care manager) discussed HF-specific ICF categories to be added to the ICF checklist. The members of the expert panel had extensive experience in medical care and rehabilitation of older patients with HF. The expert panel added to the ICF checklist 8 body function categories (heart failure-specific categories: b126, b172, b250, b45, b455, b460, b545, and b740), 2 body structure categories ((heart failure-specific categories: s140 and s150), and 6 activity and participation categories (heart failure specific categories: d155, d177, d230, d240, d420, and d855). In total, 143 categories were included in the questionnaire: 39 body function, 18 body structure, 54 activity and participation, and 32 environmental factor categories. In addition, we received open-ended responses to the question regarding the elements necessary for medical care coordination.

### Statistical analysis

We calculated percentage scores after a simple tabulation of the data. The cut-off value for each ICF category was set at 50%, based on previous studies [20, 22–30]. Next, we compared the ICF categories of care managers with medical qualifications and those with care or welfare qualifications. Data analysis was performed using the Japanese version of SPSS for Windows (version 23.0; IBM Corporation, Armonk, NY, USA), and statistical significance was set at *P* < 0.05. Demographics and percentage scores of ICF categories were evaluated between the groups using χ^2^ analysis. For the free text, we analysed the data using a text-mining method and KH Corder (Version 3. Aloha 1.7 k), a software program used for quantitative content analysis which supports Japanese text [31, 32]. The text-mining data were used to calculate frequently-used words and create a co-occurrence network. A co-occurrence network diagram presents words with similar patterns of occurrence among words with high frequency of occurrence in the text data.

Therefore, it is a network diagram in which words with a strong degree of co-occurrence are connected by lines; the stronger the co-occurrence relationship, the thicker the line, and the larger the circle for words with more frequent occurrences.

### Ethical considerations

This study was conducted in accordance with the principles of Declaration of Helsinki. We obtained approval from the Hiroshima University of Epidemiological Research Ethics Review Board (approval number: E-2217). The survey participants were informed in the survey request letter that personal information would be handled and that there would be no disadvantage if they refused to participate in the survey. They were also informed that their consent to the survey would be granted by completing and returning the survey. This study was supported by MHLW Comprehensive Research on Statistical Information Program Grant Number JPMH20AB1002.

## Results

We received 520 responses from the participants (response rate: 74.8%). Table 1 shows the basic qualifications of the care managers. The majority were care workers (348; 63.0%), followed by social workers (96; 17.4%), and nurses (38; 6.9%). The average duration of work experience was 9.6 ± 5.0 years.

**Table 1:** Basic qualifications of the participants (*n* = 520)

Basic qualifications of care managers	n	%
Medical qualifications		
Pharmacists	1	0.2
Public health nurses	11	2.0
Nurses	38	6.9
Practical nurses	12	2.2
Physiotherapists	5	0.9
Occupational therapists	2	0.4
Dietitians	6	1.1
Dental hygienist	11	2.0
Care or welfare qualifications		
Social workers	96	17.4
Psychiatric social workers	4	0.7
Certified care workers	348	63.0
Others	2	0.4
Unlfilled	16	2.9
Total	552	100.0

Note: There is overlap because some participants have double or triple licences

Forty-nine ICF categories were identified as “necessary” for care planning in older patients with HF by at least 50% of the participants: 18 body function categories, one body structure category, 21 activity and participation categories, and nine environmental factor categories (Tables 2, 3 and 4). The percentage of respondents who answered “necessary” for the 10 body function categories, 1 body structure category, and 3 activity and participation categories were significantly higher in the medical qualification group than in the welfare or care group (Tables 2, 3 and 4: The bold items). There was no significant difference in the environmental factors between the two groups.

**Table 2:** ICF categories of body function and body structure that were considered relevant by ≥50% of participants

ICF categories		Total	Medical qualifications	Welfare or care qualifications	*p*
**b110**	**Consciousness function**	**55.2%**	**69.0%**	**52.8%**	**0.025**
b114	**Orientation function**	55.4%	66.7%	53.5%	0.078
b130	**Energy and drive function**	56.2%	65.5%	54.2%	0.070
**b134**	**Sleep function**	**51.7%**	**67.8%**	**47.7%**	**<0.01**
**b164**	**Higher-level cognitive functions**	**50.2%**	**67.8%**	**47.2%**	**0.007**
b280	Pain function	73.1%	78.2%	72.4%	0.498
**b410**	**Heart function**	**78.1%**	**90.8%**	**75.1%**	**0.001**
**b415**	**Blood vessel function**	**56.5%**	**74.7%**	**52.8%**	**<0.01**
**b420**	**Blood pressure function**	**73.8%**	**89.7%**	**70.3%**	**<0.01**
**b440**	**Respiration function**	**68.3%**	**81.6%**	**66.2%**	**0.028**
**b455**	**Exercise tolerance function**	**73.1%**	**86.2%**	**71.0%**	**0.030**
b460	Sensations associated with cardiovascular and respiratory functions	76.2%	86.2%	74.6%	0.090
b525	Defaecation function	66.9%	80.5%	65.0%	0.059
**b530**	**Weight maintenance functions**	**63.1%**	**79.3%**	**60.2%**	**0.006**
**b545**	**Water, mineral and electrolyte balance functions**	**56.3%**	**77.0%**	**52.0%**	**<0.01**
b620	Urination function	81.5%	87.4%	80.8%	0.392
b710	Mobility of joint function	56.5%	58.6%	56.4%	0.865
b730	Muscle power function	56.7%	65.5%	54.9%	0.093
**s410**	**Structure of the cardiovascular system**	**54.2%**	**67.8%**	**51.3%**	**0.007**

The bold items indicate significant differences between participants with medical qualifications and welfare or care qualifications

**Table 3:** ICF categories of activity and participation that were considered relevant by ≥50% of participants

ICF categories		Total	Medical qualifications	Welfare or care qualifications	*p*
d177	Making decisions	70.0%	80.5%	68.8%	0.239
**d230**	**Carrying out daily routine**	**60.4%**	**73.6%**	**57.8%**	**0.015**
**d240**	**Handing stress and other psychological demands**	**55.2%**	**70.1%**	**51.8%**	**0.002**
d310	Communicating with-receiving-spoken messages	60.6%	72.4%	58.8%	0.087
d330	Speaking	54.2%	63.2%	52.8%	0.075
d350	Conversation	63.8%	71.3%	62.8%	0.332
d420	Transferring oneself	58.3%	65.5%	57.1%	0.266
d450	Walking	86.3%	86.2%	86.3%	0.725
d510	Washing oneself	64.4%	70.1%	63.3%	0.286
d520	Caring for body parts	51.3%	54.0%	50.6%	0.493
d530	Toileting	77.9%	82.8%	77.2%	0.461
d540	Dressing	62.9%	66.7%	61.9%	0.335
d550	Eating	81.9%	86.2%	80.8%	0.187
d560	Drinking	77.5%	78.2%	77.0%	0.567
**d570**	**Looking after one’s health**	**76.5%**	**85.1%**	**74.6%**	**0.034**
d620	Acquisition of goods and services	53.5%	62.1%	52.3%	0.276
d630	Preparing meals	63.8%	70.1%	62.8%	0.332
d640	Doing housework	62.3%	64.4%	62.1%	0.852
d710	Basic interpersonal interactions	62.7%	73.6%	61.2%	0.113
d760	Family relationships	74.8%	81.6%	73.6%	0.210
d920	Recreation and leisure	50.4%	52.9%	50.1%	0.808

The bold items indicate significant differences between participants with medical qualifications and those with welfare or care qualifications

**Table 4:** ICF categories of environmental factors that were considered relevant by ≥50% of participants

ICF categories		Total	Medical qualifications	Welfare or care qualifications	*p*
e310	Immediate family	89.8%	89.7%	90.2%	0.585
e315	Extended family	66.7%	66.7%	67.1%	0.686
e320	Friends	66.5%	66.7%	66.7%	0.901
e325	Acquaintances, peers, colleagues, neighbours, and community members	61.3%	66.7%	61.2%	0.854
e340	Personal care providers and personal assistants	53.3%	51.7%	53.7%	0.680
e355	Health professionals	58.7%	64.4%	57.1%	0.141
e410	Individual attitudes of immediate family members	73.7%	74.7%	73.9%	0.829
e575	General social support services, systems, and policies	59.8%	63.2%	60.0%	0.893
e580	Health services, systems, and policies	58.5%	58.6%	59.2%	0.473

**Fig. 1:**
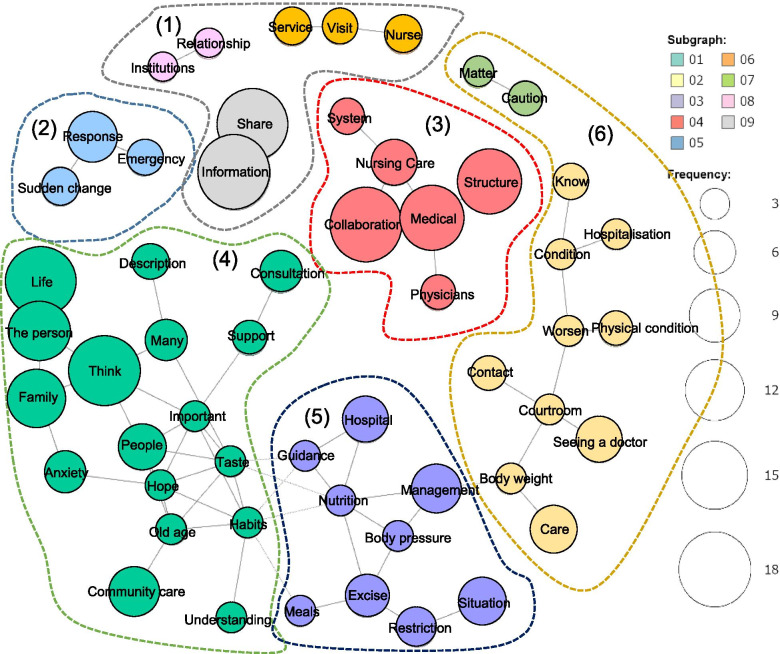
Co-occurrence network of words: necessary elements for medical care coordination. The size of the circle indicates the number of times the word appears. Word-to-word connections indicate the strength of the connections before and after the free text

Regarding the co-occurrence network, the necessary elements for medical care coordination were classified into nine subgraphs (Fig. 1). The most frequently used words were “Information”, “Share”, “Collaboration”, “Life” and “Think”. We classified these nine subgraphs into six subgroups: (1) information sharing with related organisations; (2) emergency response; (3) a system of cooperation between medical facilities and care facilities; (4) consultation and support for the thoughts and concerns of the patient and family about life, (5) management of nutrition, exercise, blood pressure, and other factors, (6) guidelines for consultation and hospitalisation when physical conditions worsen.

## Discussion

### Clarification of ICF categories for care management

In this survey, we found that 49 ICF categories were necessary for care managers to develop a care plan for older patients with HF. The ICF categories in this study matched the 19 categories of the ICF core set for chronic ischaemic heart disease in a previous study [23]. Furthermore, 43 ICF categories in this study were consistent with those in our previous study of cardiac rehabilitation instructors [16]. One explanation for the difference between the ICF core set for chronic ischaemic heart disease and the results of the present study is that the ICF categories specific to older people may have been selected because the present study included older patients with HF. In contrast, approximately 90% of the ICF categories in this study matched those selected by the cardiac rehabilitation instructors in our previous study. Therefore, we suggest that the 43 matching ICF categories are necessary for information sharing for medical and care coordination.

### Differences in body functions assessment between medical and welfare professionals

There was a significant difference in the ICF categories considered necessary between medical and welfare or care professionals. Since about 80% of the care support professionals in Japan are care workers, and the participants of this study had similar proportions, it is reasonable to assume that the results of this study reflect the nationwide trend in Japan. In the ICF categories of body function and health care, those in the medical qualifications group were found to have a higher awareness of assessment than those in the welfare or care qualifications group. Lack of medical knowledge among care managers is seen as a problem when sharing information with visiting nurses and home physicians [33]. From the results of this study, we suggested that medical terminologies should be explained in an easy-to-understand manner when proposing a care plan to a care manager with a welfare/care licence. In addition, education on the management of diseases, such as HF, for care managers with welfare care qualifications is an issue [34].

### Necessary elements for collaboration between medical and welfare/care professionals

The results of the co-occurrence network show that care managers need an information sharing system for medical and care, community networks, self-management to prevent heart failure exacerbations, decision-making support for patients and families, and a crisis plan (e.g., emergency response, guidelines for hospitalization, etc.). The structure of the co-occurrence network is very similar to the framework of Wagner’s chronic care model (CCM) [35, 36]. The A CCM-based approach to chronic diseases, including heart failure, has been shown to be effective in improving medication management, quality of life, preventing readmission and healthcare costs [37–41]. Furthermore, the CCM is one of the models for a comprehensive community care system in Japan. We propose that an information sharing system for medical and care collaboration include not only the 49 ICF categories for older patients with heart failure but also the contents indicated by the co-occurrence network. In future studies, we desire to construct an information-sharing system between medical and nursing care that incorporates the 49 ICF items identified in this study and the elements necessary for the collaboration demonstrated in the co-occurrence network, and examine its effectiveness in preventing rehospitalisation for heart failure.

### The approaches to prevent exacerbations in elderly patients with heart failure based on the results of this study

Based on the results of this study, we have several suggestions for preventing rehospitalisation in elderly patients with heart failure. The ESC guidelines recommend the management of chronic heart failure with multidisciplinary interventions [42]. In hospitals, multidisciplinary teams consisting of physicians, nurses, pharmacists, dietitians, physical therapists, occupational therapists, and social workers are responsible for ICF assessment and disease education and self-monitoring. In the community, care managers, visiting nurses, and visiting physical or occupational therapists are responsible for self-monitoring practices and disease management, including telemonitoring. A common language using ICF is effective as an information sharing system between the hospital and the community. Through the Hiroshima Prefecture Heart Health Promotion Project, we aim to provide seamless multidisciplinary team interventions that include patient and family needs and decision support, using ICF to share information consistently from admission to post-discharge [12].

### Limitations

This study has several limitations. First, The ICF categories investigated in this study do not include individual factors. Personal factors include age, gender, values, lifestyle, coping, and personality. Since patient-centred intervention is the principle in the care of chronic diseases [40], it is necessary to include personal factors when developing an ICF information sharing system. Second, because our study was conducted with care managers in Hiroshima Prefecture, it is difficult to generalise the results to HF care facilities throughout Japan or overseas. Third, in this survey, more than 80% of the respondents were in the welfare/care workers, and it is undeniable that there is occupational bias in the responses. Fourth, since patients and family physicians were not included in this study, the information needed for home care is not sufficiently comprehensive. Fifth, since a questionnaire survey for professionals was used, the appropriateness of the selected ICF categories should be verified using empirical data. In future, we plan to conduct a survey study on information needed for home care for patients and family physicians. In addition, it will be necessary to establish an ICF evaluation method that can be commonly used by medical and care providers, and to build an effective information-sharing system in regional networks.

## Conclusions

The findings of our study showed that 49 ICF categories were rated as necessary by care managers for care planning, and there was a significant difference in perception between care managers in medical professions and those in welfare professions. In the future, we wish to develop an information sharing system between medical professionals and care managers that includes these 49 ICF categories to prevent rehospitalisation of elderly patients with heart failure.

## Data Availability

The datasets used and/or analysed during the current study are available from the corresponding author on reasonable request.

## Abbreviations

ICF: International Classification of Functioning, Disabilities, and Health
HF: Heart failure
